# Supplementary material for: Ni-Based Catalyst Derived from NiAl Layered Double Hydroxide for Vapor Phase Catalytic Exchange between Hydrogen and Water
Source: Nanomaterials (Basel). 2019 Nov 25;9(12):1688. doi: 10.3390/nano9121688 (PMC6956106; doi:10.3390/nano9121688)
Supplement: Supplementary file 1 [file nanomaterials-09-01688-s001.pdf]

nanomaterials

Supplementary Information

Ni-Based Catalyst Derived from NiAl Layered Double Hydroxide for Vapor Phase Catalytic Exchange between Hydrogen and Water

Xiaoyu Hu [1,2,†], Peilong Li [2,†], Xin Zhang [2], Bin Yu [2], Chao Lv [2], Ning Zeng [2], Junhong Luo [2], Zhi Zhang [2], Jiangfeng Song [2,*] and Yong Liu [1,*]

1 School of Resource Environmental and Safety Engineering, University of South China, Hengyang 421001, Hunan, China; hu822066398@163.com
2 Institute of Materials, China Academy of Engineering Physics, Jiangyou 621908, Sichuan, China; lipeilong2012@126.com (P.L.); a753951_xin@163.com (X.Z.); yubincaep@163.com (B.Y.); lvchao219@foxmail.com (C.L.); 15882895785@139.com (N.Z.); luojunhong@caep.cn (J.L.); zhangzhi@caep.cn (Z.Z.)
* Correspondence: iterchina@163.com (J.S.); dtscichina@126.com (Y.L.); Tel.: +86-13990105175 (J.S.); +86-13507341111 (Y.L.)
† These authors contributed equally to this work.

Contents

1. Supplementary Experimental method and corresponding Figures S1-S3 and Table S1

2. Supplemental SAED patterns of samples

3. Supplemental properties of XPS spectra for catalysts before and after reaction

4. Supplemental X-ray diffraction patterns of the tested NiAl-LDO

5. Supplemental X-ray diffraction patterns of the tested NiO/γ-Al_2O_3

1. Supplementary Experimental method and corresponding Figures S1-S3 and Table S1

1.1. Preparation of NiAl-LDH with different synthesis conditions

All reagents used in this work were of analytical grade and used without further purification. The synthesis conditions were optimized by adjusting the molar ratio of Ni/Al/precipitator (or Urea) from 2/1/7 to 2/1/14, the aging time from 6 h to 36 h and the hydrothermal temperature from 110 °C to 150 °C. The acronyms with the molar ratio of constituents and the temperature of hydrothermal treatment as well as time of hydrothermal treatment were applied to denote the prepared catalysts' precursor. The x in NiAl-x denotes the only changed synthesis conditions. For instance, NiAl-2/1/7 indicates the NiAl-LDHs were synthesized in condition with the 2/1/7 of constituent molar ratio and hydrothermal treatment for 12 h at 150 °C. NiAl-6 h donates NiAl-LDHs were prepared in condition with the 2/1/14 of constituent molar ratio and hydrothermal treatment for 4 h at 150 °C. NiAl-110 °C represents the NiAl-LDHs were synthesized constituent molar ratio of 2/1/14 and hydrothermal treatment for 12 h at 110 °C.

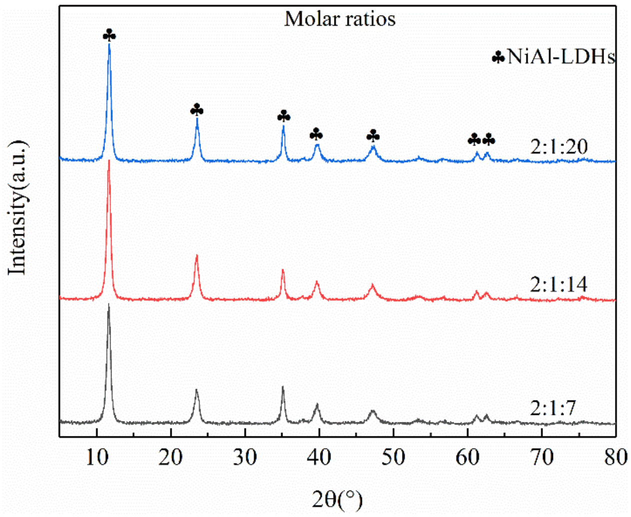

Figure S1. X-ray diffraction patterns of NiAl-LDH under different Ni/Al/Urea molar ratios.

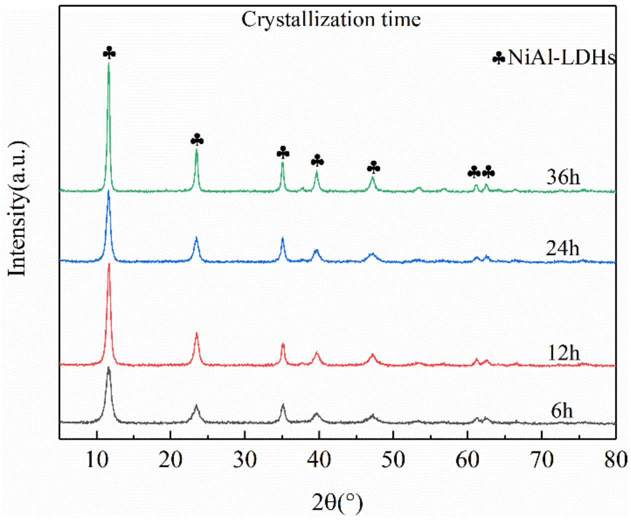

Figure S2. X-ray diffraction patterns of NiAl-LDH under different crystallization time.

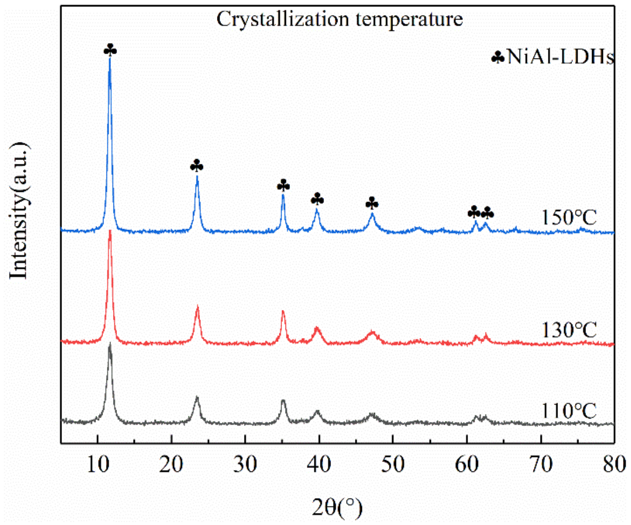

Figure S3. X-ray diffraction patterns of NiAl-LDH under different crystallization temperatures.

1.2. Analysis table of XRD patterns

Table S1. Analysis of XRD patterns for NiAl-LDH prepared by hydrothermal time, adjusting hydrothermal temperature as well as Ni/Al/Urea molar ratio.

Sample	(003) integral intensity	$W_{1/2}$ for (003)	$d_{(003)}$	Crystallite size[a]	Lattice parameter	
	(a.u.)	(°)	(nm)	(nm)	a (nm)	c (nm)
NiAl-2/1/7	12011	0.540	0.759	12.0	3.024	22.809
NiAl-2/1/14	14012	0.539	0.758	12.5	3.025	22.811
NiAl-2/1/20	12053	0.553	0.756	12.3	3.023	22.749
NiAl-6 h	9436	0.699	0.760	11.7	3.024	22.841
NiAl-12 h	14012	0.539	0.758	12.5	3.025	22.811
NiAl-24 h	13875	0.541	0.761	11.6	3.025	22.866
NiAl-36 h	12434	0.368	0.760	21.9	3.028	22.846
NiAl-110°C	7350	0.654	0.758	7.8	3.023	22.825
NiAl-130°C	10930	0.648	0.756	10.0	3.022	22.779
NiAl-150°C	14012	0.539	0.758	12.5	3.025	22.811

[a] Crystallite size calculated as XRD coherent domain in (003) direction using Scherrer's formula.

2. Supplemental SAED patterns of samples

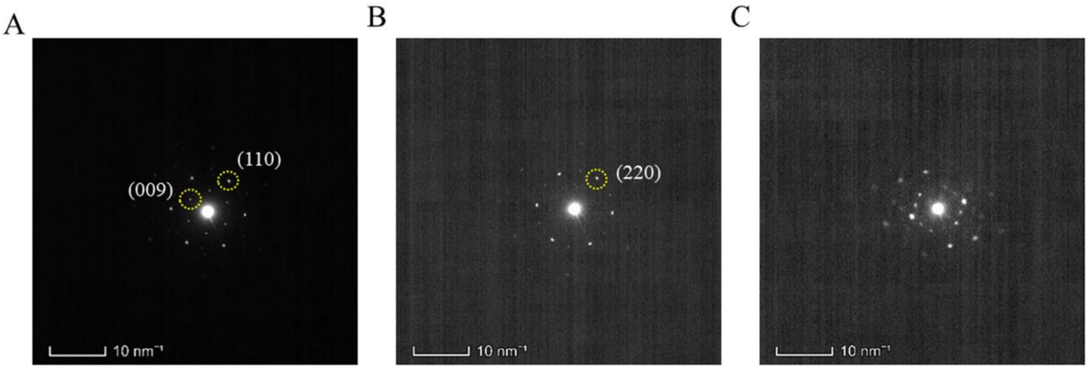

Figure S4. SAED patterns of samples: A) NiAl-LDH; B) NiAl-LDO; C) NiO/γ-Al_2O_3.

3. Supplemental properties of XPS spectra for catalysts before and after reaction

Table S2. Properties of XPS spectra for catalysts before and after reaction.

Catalysts	Before reaction		After reaction	
	Ni species	Relative intensity (%)	Ni species	Relative intensity (%)
NiAl-LDO	Ni^{2+}	100	Ni^{2+}, Ni^0	97.86, 2.14
NiO/γ-Al_2O_3	Ni^{2+}	100	Ni^{2+}, Ni^0	62.16, 37.84

4. Supplemental X-ray diffraction patterns of the tested NiAl-LDO

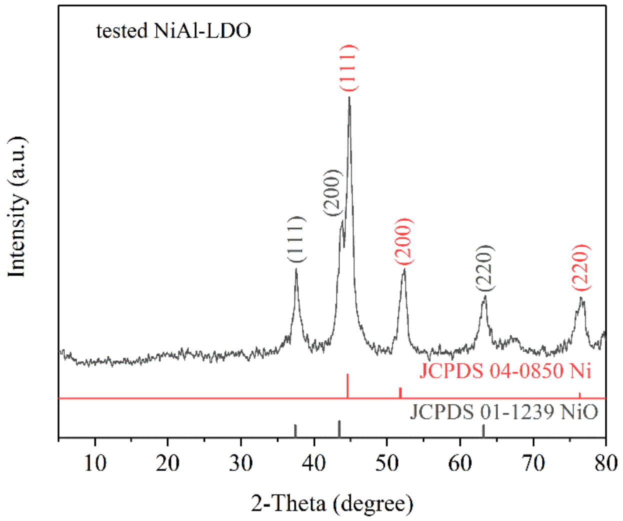

Figure S5. X-ray diffraction patterns of the tested NiAl-LDO. (Without pre-reduction, after reaction under 500 ℃.).

5. Supplemental X-ray diffraction patterns of the tested NiO/γ-Al_2O_3

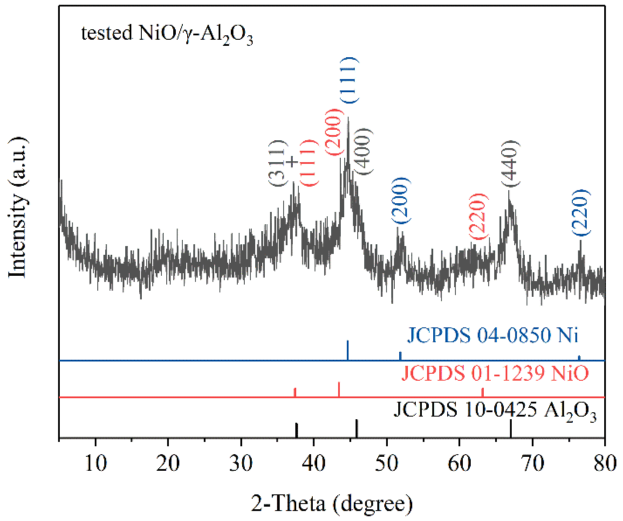

Figure S6. X-ray diffraction patterns of the tested NiO/γ-Al_2O_3. (Without pre-reduction, after reaction under 500 ℃.).
